# Some Diseases of the Male Genital System

**Published:** 1908-02-08

**Authors:** 


### SOME DISEASES OF THE MALE GENITAL SYSTEM.

### VII.—THE TREATMENT OF RETAINED TESTIS.

The question of the treatment of retained testis is one which is almost as much open to discussion as is the causation of the condition. One thing, however, is certain, and that is that no palliative measures are of any avail. Even in recent times there were some who placed their reliance on the application of a special truss whose pad was made of wood, designed more or less in the shape of a horseshoe, so that the testis could be brought down into the scrotum and retained there without pressure on the constituents of the cord. One obvious disadvantage lies in the fact that it is only in rare cases that a retained testis can be manipulated into the scrotum, and, even if it can, the apparatus does not ever effect a cure; for as soon as the truss is removed the testis is again withdrawn. In fact, it is no exaggeration to say that it is less harmful to leave things as they are than to employ this form of truss, whose use has now been entirely superseded by other methods.

Of course, if the testis is completely retained within the abdomen the patient may be quite unconscious of any abnormality except the fact that one testis is absent from the scrotum; that is to say the condition may cause him no pain nor inconvenience, and in this case it may be wise to leave things well alone. The only danger in doing so is that if acute orchitis supervenes on an attack of gonorrhœa peritonitis may be the result, and, although this is rare, the patient should be warned of the danger of exposing himself to infection. The argument that a retained testis is more liable to malignant disease than one which is in its normal situation, and should, therefore, be removed at all costs, is not convincing. The greater frequency of malignant disease in the misplaced organ is extremely small, even if the collected statistics are correct, and there is not enough evidence to justify the removal of any retained testis on suspicion.

If, however, the testis is retained in relation to the inguinal canal, the only rational treatment is to perform an operation for its conveyance to the scrotum; and if during the course of the operation this be found impossible, the alternatives which face the surgeon are (i.) replacement of the testis in the abdominal cavity, or (ii.) castration. The advantage of operating is that the sac of the congenital hernia which is nearly always co-existent with a retained testis can be dealt with at the same time.

#### The Ordinary Operation.

The steps of the operation in the early stages are identical with those of the operation for radical cure of an inguinal hernia. As soon as the testis and the structures of the cord are found and isolated, the first manœuvre is to separate the hernial sac and ligature it at its upper extremity near the internal abdominal ring. The sac can now be removed by cutting its lower attachment as near as possible to the testis; it is not necessary to ligature its lower end and so form a false tunica vaginalis. Now comes the all-important question, are the structures of the cord long enough to allow the testis to come down into the scrotum? If not, they may all be cut with the exception of the vas. It is a curious fact that reposition of the testis in the scrotum is rarely, if ever, resisted by shortness of the vas. It is the vessels of the cord which cause trouble. They may be cut without any fear that the testis will have an insufficient blood supply. The vessels which surround the vas itself will be quite adequate to carry on its circulation.

Another difficulty is due to the fact that the corresponding half of the scrotum is ill developed, and often incapable of accommodating a testis to whose presence it has been unaccustomed. It is not sufficient to bring the testis down into the scrotum and sew up the wound. If this is done the organ will be retracted to its original position in a very short time. Therefore, it must be mechanically fixed in its new position. This may be done by passing silk sutures through the tunica albuginea and through the scrotum, and then either fixing them to the skin of the thigh or to a wire frame which is specially devised to fit the front of the thigh, and is incorporated in the dressings. The sutures may be removed in about 20 days, and in a favourable case it will be found that there is no further tendency on the part of the testis to retract. If the operation is successful, it is said that the testis may begin to undergo development in its new surroundings. On the other hand, there are some who say that the handling and manipulation required to bring the testis down into the scrotum may determine the onset of degenerative changes, and that no benefit accrues as the result of such procedures.

If during the course of the operation it is found that even after cutting the vessels of the cord the testis cannot be brought down, it may either be replaced in the abdomen behind the peritoneum or removed altogether. The former method should be chosen if the testis does not appear to be quite atrophic and functionless; but should degenerative changes have set in which have rendered the organ valueless, there need be no hesitation in performing castration forthwith.

Most of the complications which beset the testis in an abnormal position call for its removal—*e.g.* malignant disease or necrosis, as the result of torsion; and even if the only complication is inflammation, it is perhaps better to remove the testis at once than to watch the patient on the chance of the inflammation subsiding spontaneously.

So far these remarks have referred to the treatment of a case in which only one testis is retained, while the other occupies its normal position. But if, as is not infrequently the case, the condition is bilateral, the question of treatment is more difficult to decide. In view of the importance to the individual of the internal secretion of the testis, removal of one testis must never be undertaken lightly, lest the other one is functionless; and, further, should operation be decided on, one side should be done first and a long interval be allowed to elapse before the second testis is brought down, and then this must only be done if the result of the first operation is satisfactory.

